# Estimating Leaf Area Index in Row Crops Using Wheel-Based and Airborne Discrete Return Light Detection and Ranging Data

**DOI:** 10.3389/fpls.2021.740322

**Published:** 2021-11-29

**Authors:** Behrokh Nazeri, Melba M. Crawford, Mitchell R. Tuinstra

**Affiliations:** ^1^Lyles School of Civil Engineering, Purdue University, West Lafayette, IN, United States; ^2^Department of Agronomy, Purdue University, West Lafayette, IN, United States

**Keywords:** high-throughput phenotyping, remote sensing, LiDAR, leaf area index, machine learning, row crops

## Abstract

Leaf area index (LAI) is an important variable for characterizing plant canopy in crop models. It is traditionally defined as the total one-sided leaf area per unit ground area and is estimated by both direct and indirect methods. This paper explores the effectiveness of using light detection and ranging (LiDAR) data to estimate LAI for sorghum and maize with different treatments at multiple times during the growing season from both a wheeled vehicle and Unmanned Aerial Vehicles. Linear and nonlinear regression models are investigated for prediction utilizing statistical and plant structure-based features extracted from the LiDAR point cloud data with ground reference obtained from an in-field plant canopy analyzer (indirect method). Results based on the value of the coefficient of determination (*R*^2^) and root mean squared error for predictive models ranged from ∼0.4 in the early season to ∼0.6 for sorghum and ∼0.5 to 0.80 for maize from 40 Days after Sowing to harvest.

## Introduction

Determination of Leaf Area Index (LAI) is essential for modeling the interaction between the atmosphere and the biosphere (Zhu et al., 2020). It is an important biophysical parameter that acts as a primary control for energy, water, and gas exchange within a vegetated ecosystem (Jensen et al., 2008; Zheng and Moskal, 2009). Estimation of LAI is also important for crop modeling (Lobell et al., 2015; Akinseye et al., 2017) and plant breeding (Blancon et al., 2019). Both direct and indirect approaches have been investigated to estimate LAI. Direct methods, which are based on measuring the area of the leaves directly, are accurate but costly, labor-intensive, and time-consuming. In destructive sampling, plants are defoliated within a specific area, and the one-sided leaf surface area is measured from imagery or with an electronic area meter (White et al., 2019) such as an LI-3100C. The average leaf biomass fraction and specific leaf weight, which is defined as leaf dry weight (the oven-dry mass), divided by the one-sided area of the fresh leaves are used to compute LAI, for each plot and sampling date (Yang et al., 2021).

Indirect optical methods estimate LAI from the canopy gap fraction that is defined as the effective LAI (LAI_eff_). The relationship between LAI_eff_ and true LAI derived from a direct method, which assumes that the leaves are randomly distributed within the canopy, is shown in Eq. 1 (Chen et al., 2005; Ryu et al., 2010).


(1) $${\text{LAI}}_{\text{eff}}\,\left(\theta \right)=\Omega \,\left(\theta \right)\times \text{LAI}$$

where Ω(θ) is the canopy clumping index that describes the non-randomness of the leaf foliage distribution; it can be estimated through the nonrandom distribution of gap fractions using the logarithmic gap fraction averaging method, and θ is the solar zenith angle (Fang et al., 2019).

Digital cover photography, digital hemispherical photography, and the LAI-2200C plant canopy analyzer are all used to obtain indirect optically-based estimates of LAI (Fournier and Hall, 2017; Fang et al., 2019). Direct measurement methods and some optical methods are also used as references for indirect measurement techniques (Richardson et al., 2009). Indirect methods have been developed for determining LAI over large areas using both active and passive remote sensing. Within the last decade, light detection and ranging (LiDAR) has been used for mapping, modeling, and spatial analysis in many applications, including estimation of LAI. The advantage of LiDAR compared to other remote sensing technologies is that it directly provides three-dimensional coordinates. Promising results have been obtained from LiDAR (Jimenez-Berni et al., 2018) and in combination with hyperspectral imagery (Masjedi et al., 2018, 2019) in modeling biophysical characteristics, including vegetation height and above-ground biomass for agriculture applications (Nie et al., 2016; ten Harkel et al., 2020). LiDAR has also been used to model forest canopy structure (Lefsky et al., 2002) and to estimate LAI in forests (Zhao and Popescu, 2009; Korhonen et al., 2011; Jung and Crawford, 2012; Alonzo et al., 2015).

To estimate LAI from LiDAR, empirical models are developed to represent the relationship between the ground reference LAI and LiDAR-derived metrics. Two types of LiDAR metrics are commonly used in LAI prediction, the Beer-Lambert law based on the laser penetration index (LPI; Richardson et al., 2009) and allometric measurements that are statistically-based features (Pope and Treitz, 2013). Allometric-related features include the mean height and standard deviation, maximum height of all returns, and the coefficient of variation of height. Features based on the Beer-Lambert law include gap fraction and LPI (Nie et al., 2016). Pope and Treitz (2013) demonstrated the combined use of airborne discrete return LiDAR data and WorldView-2 high-resolution imagery to predict LAI in a boreal mixed wood forest. Digital hemispherical photos were used as a ground reference, and statistically significant LiDAR-based inputs for a stepwise linear regression model included the ratio of the first return and total return, the vertical distribution ratio, crown closure, and a vertical complexity index (VCI) that represents structural homogeneity with height (Ludwig et al., 1988; van Ewijk et al., 2011; Pope and Treitz, 2013).

Few studies have focused on estimating LAI for row crops, such as maize, e.g., Nie et al. (2016) and sorghum, e.g., Lang (1986). In addition, in most remote sensing focused studies, discrete return LiDAR data are acquired by manned aircraft and Unmanned Aerial Vehicles (UAVs), which have lower point density and laser penetration than ground-based platforms. Ground-based LiDAR data can acquire data at a very high spatial resolution over shorter crops compared to airborne platforms, and depending on the plant structure, can potentially penetrate deeper into the canopy. Further, these platforms are not subject to localized changes in position, elevation, and look angle that are common with airborne platforms, but are restricted to operation in field conditions during which they can drive and collect data.

Nazeri (2021) investigated the destructive sampling method as a ground reference in estimation of LAI from LiDAR acquired by a UAV over a sorghum field experiment. Three sets of ground reference data collected by the Purdue team in 2019 to parameterize a crop growth model were provided as ground reference data. The relationship between the LiDAR data and LAI computed using destructively sampled ground reference data was weak. The results were not unexpected, as the LiDAR data are physically more closely related to the gap fraction than the assumptions for LAI calculations based on destructive sampling (Hammer et al., 2010; Fang et al., 2019; Yang et al., 2021). The low *R*^2^ of models obtained using the destructive sampling ground reference, coupled with the practical limitations for performing extensive destructive sampling through the season motivated this study of an indirect ground reference method coupled with extensive data acquisitions during the 2020 growing season.

This paper is an exploratory study of LAI prediction using LiDAR point cloud data acquired by a converted high-clearance tractor/sprayer with a custom sensor boom and by low altitude UAVs over sorghum and maize plant breeding experiments. LiDAR platforms and systems with different laser units were evaluated at multiple altitudes for obtaining LAI. Remote sensing acquisitions were matched to the field-based LAI measurements using near-coincident data acquisitions. Multiple strategies for feature extraction were investigated for developing regression-based predictive models, including stepwise multiple linear regression (SMLR), partial least squares regression (PLSR), and support vector regression (SVR). The predictive models were developed based on the indirect ground reference method and evaluated based on the resulting *R*^2^ values and the root mean squared error of the residuals. Contributions of the study include investigation of multiple LiDAR-based features for multitemporal prediction of LAI via regression models and evaluation of the capability of LiDAR sensors and platforms for acquiring data to predict sorghum and maize LAI at multiple times during the growing season.

## Materials and Methods

### Study Area and Experiment Setting

**FIGURE 1:**
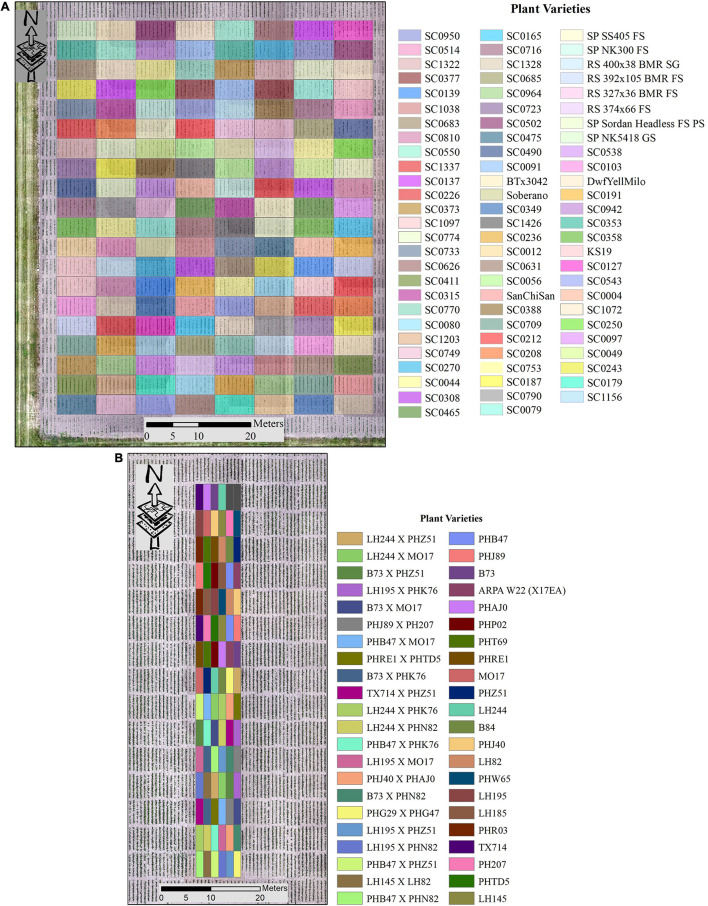
Plot variety layout **(A)** SbDivTc_Cal and **(B)** HIPS.

The experiments for this study were conducted at the Agronomy Center for Research and Education at Purdue University, West Lafayette, IN, United States, to evaluate the potential of sorghum varieties for biomass production. Both ground reference and LiDAR data were acquired during the 2020 growing season. In this study, near concurrent ground-based and UAV LiDAR data were analyzed. The LiDAR data were collected from the Sorghum Biodiversity Test Cross Calibration Panel (SbDivTc_Cal) and the maize High-Intensity Phenotyping Sites (HIPS) Panel. The SbDivTc_Cal experimental design included 80 varieties two replicates in a randomized block design planted in 160 plots (plot size: 7.6 m × 3.8 m), ten rows per plot (row number is counted from the west to east). All 160 plots were included in the analysis for the SbDivTc_Cal data, as LAI ground reference data were acquired for all the plots in the experiment. The HIPS maize experiment contained 44 varieties of maize with two replicas, including hybrids and inbreds. This experiment had 88 plots (plot size: 1.5 m × 5.3 m), two rows per plot. In the early stages, sorghum and maize have very similar plant structures, although sorghum is planted at a higher density (∼200,000 plants/hectare) compared to maize (∼75,000 plants/hectare). During the growing season, the geometric structure of sorghum becomes more complex as tillers develop, decreasing canopy penetration. Figure 1 shows the layout of the SbDivTc_Cal and HIPS plots based on the respective genotypes.

**FIGURE 2:**
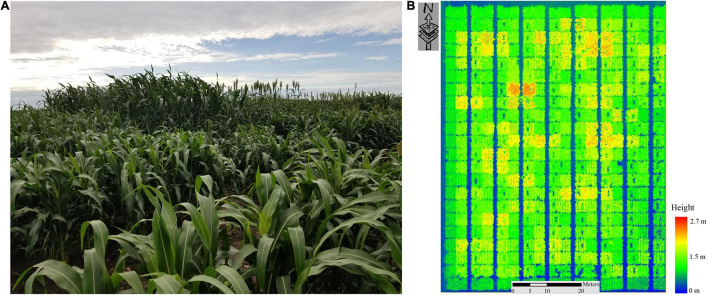
**(A)** Photograph of the SbDivTc_Cal panel (7/20/2020), **(B)** LiDAR-Based Height Map of SbDivTc_Cal Sorghum Panel (7/20/2020).

Differences between varieties can be seen clearly in terms of physical characteristics shown in a photo (Figure 2A), and in the LiDAR-based height map acquired by a UAV on 7/20/2020, 68 days after sowing (DAS; Figure 2B).

### Field Ground Reference Data

**FIGURE 3:**
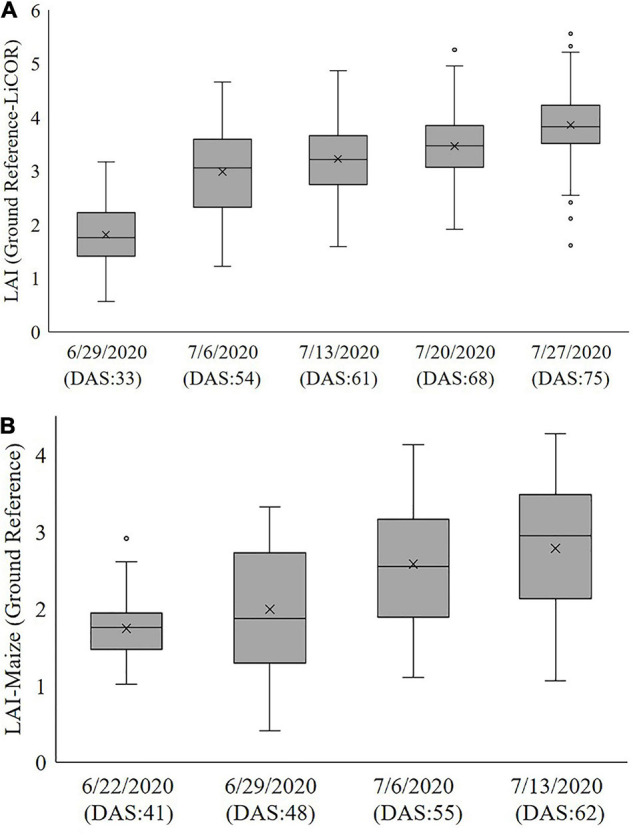
Measured ground reference LAI of **(A)** SbDivTc_Cal and **(B)** HIPS using LAI–2200C (2020).

In 2020, reference data were collected weekly from June 29 to July 27 for sorghum and from June 22 to July 13 using a handheld plant canopy analyzer (LAI-2200C). The LAI-2200C is a portable instrument for acquiring an indirect measurement of LAI_eff_ based on canopy gap fraction analysis (Welles and Cohen, 1996; Sonnentag et al., 2007; Černý et al., 2019). In sorghum, to avoid the impact of adjacent plots and destructive sampling, LiDAR data from Rows 2 and 3 of each plot were associated with each reference value for developing the predictive models. Two sets of five measurements one measurement above the canopy and four measurements below the canopy near the ground between rows 2 and 3 in the direction of the rows (north-south) were made according to the recommended protocol, then a representative value per plot was calculated using the Field Viewer 2200 (FV2200) software. These values were used as the primary reference data for developing predictive models of LAI based on the LiDAR remote sensing data. The ground reference values ranged from 0.5 to 6 for sorghum and 0.5 to 5 for maize, increasing during the period of the growing season until sampling was stopped after flowering. The box plots in Figure 3 show the range of values of ground reference data for both crops within ±1.96 standard deviations for the LAI–2200C based on the date of data collection and corresponding DAS. The values of LAI exceeding 95% were from photoperiod sensitive varieties, whose characteristics increasingly differ from the rest of the experiment as the season progresses. The sequence of 2020 plant canopy analyzer data was used as a ground reference for evaluating the LiDAR-based metrics. Remotely sensed LiDAR data and ground reference acquisitions were separated by no more than 3 days. Table 1 summarizes the experiment over the SbDivTc_Cal and HIPS 2020 experiments.

**TABLE 1:** Experimental design for the 2020 growing season.

Experiment	Genotype	# of plots	# of varieties	Sowing date	Harvest date
HIPS	Hybrid/inbred	88	44	May 12	October 1
SbDivTc_Cal	Hybrid	160	80	May13	August 15

### Light Detection and Ranging Point Cloud Data Acquisitions

#### Platforms and Sensors

**FIGURE 4:**
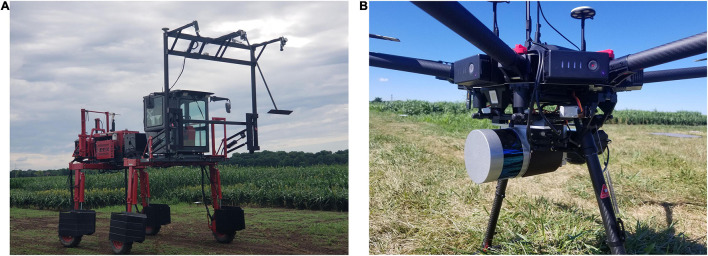
**(A)** PhenoRover platform with RGB/LiDAR/Hyperspectral/GNSS/INS sensors, **(B)** UAV-2 with RGB/LiDAR/GNSS/INS sensors in 2020.

Remote sensing data were collected by the UAV weekly, first prior to planting to develop the baseline terrain model and at intervals of 1–2 weeks thereafter, depending on the weather, throughout the growing season. Two M600P UAVs were flown over the study area at altitudes of 20 and 40 m and speeds of 3–5 m/s. The UAVs were equipped with a Velodyne VLP-Puck LITE and a Velodyne VLP-32C, respectively. The Velodyne VLP-Puck LITE has 16 channels that are aligned vertically from −15° to +15°, resulting in a total vertical field of view (FOV) of 30°. The point capture rate in single return mode is ∼300,000 points per second. The range accuracy is typically ±3 cm, with a maximum measurement range of 100 m (Velodyne VLP-Puck Lite, 2020). The Velodyne VLP-32C has 32 channels that are aligned vertically from −15° to +25°, in a total vertical FOV of 40°. The point capture rate in a single return mode is ∼600,000 points per second. The range accuracy is typically ±3 cm, with a maximum measurement range of 200 m (Velodyne VLP-32C, 2020). The UAVs were equipped with an integrated global navigation satellite system/inertial navigation system (GNSS/INS) Trimble APX-15v3 for direct georeferencing (Hasheminasab et al., 2020). LiDAR data were acquired by a wheel-based system, a LeeAgra Avenger agricultural high-clearance tractor/sprayer with a custom boom and mounted sensors, referred to in this study as the PhenoRover, on an experimental basis. The boom is constructed from 2.75 m wide T-slot structural aluminum, and the top of the boom can be raised to a maximum of 5.5 m height from the ground. Sensors mounted on the boom include a Headwall hyperspectral VNIR machine vision camera, two FLIR RGB cameras, and a Velodyne VLP-Puck Hi-Res LiDAR, as well as the GNSS/INS navigation system. The VLP-Puck Hi-Res has similar sensor specifications to the VLP-Puck LITE. Its FOV is −10° to +10° (Velodyne VLP-Puck Hi-Res, 2020). The platform speed in the field was 1.5 miles per hour. Figure 4 shows the PhenoRover and UAV platforms for the 2020 data collection. PhenoRover data were acquired limited times in 2020, subject to field conditions. Table 2 details the platforms and their mounted sensor specifications for the 2020 data collection.

**TABLE 2:** Platforms and mounted sensors specification in 2020.

Platform	Sensor	Unit	Description
**UAV-1**			
	RGB camera	1	36.4 MP Sony Alpha 7R (ILCE-7R)
	LiDAR sensor	1	Velodyne VLP 16-Puck LITE-range accuracy of ±3 cm
	GNSS/INS	1	Trimble APX-15 v2
	Hyperspectral Camera	1	Nano Hyperspectral (VINIR)
**UAV-2**			
	RGB camera	1	36.4 MP Sony Alpha 7R (ILCE-7R)
	LiDAR sensor	1	Velodyne VLP 32-range accuracy of ±3 cm
	GNSS/INS	1	Trimble APX-15 v2
**PhenoRover**			
	RGB camera	2	9.1 MP FLIR Grasshopper3 GigE
	Hyperspectral camera	1	Headwall Machine Vision 270 band line-scanning with 4.8 mm lens
	LiDAR sensors	1	Velodyne VLP-Puck Hi-Res
	GNSS/INS	1	Applanix POS-LV 125

Table 3 summarizes the LiDAR data collection and the corresponding ground reference measurements in terms of DAS relative to the data collection dates and ground reference measurements.

**TABLE 3:** Days after sowing (DAS) relative to the available ground reference and LiDAR data in two experiments over SbDivTc_Cal and HIPS.

Experiment	Platform	Flying height	Sowing date	LiDAR data collection date	DAS^1^	Ground reference date	DAS^2^
HIPS	UAV-1	N/A	05/12	06/25	44	06/22	41
	PhenoRover	N/A		06/26	45	06/29	48
	UAV-2	20 m		07/07	56	07/06	55
	UAV-1	20 m		07/11	60	07/13	62
	UAV-2	20 m		07/11	60	07/13	62
	UAV-2	20 m		07/13	62	07/13	62
	PhenoRover	N/A		07/13	62	07/13	62
SbDivTc_Cal	PhenoRover	N/A	05/13	06/26	44	06/29	47
	UAV-1	40 m		07/02	50	06/29	47
	UAV-2	20 m		07/07	55	07/06	54
	UAV-2	20 m		07/13	61	07/13	61
	UAV-1	40 m		07/17	65	07/20	68
	PhenoRover	N/A		07/20	68	07/20	68
	UAV-1	40 m		07/20	68	07/20	68
	UAV-2	20 m		07/20	68	07/20	68
	UAV-1	40 m		07/28	76	07/27	75
	UAV-2	20 m		07/28	76	07/27	75

*DAS^1^: DAS with respect to data collection data; DAS^2^: DAS with respect to ground reference data.*

#### PhenoRover and Unmanned Aerial Vehicle Light Detection and Ranging Data

**FIGURE 5:**
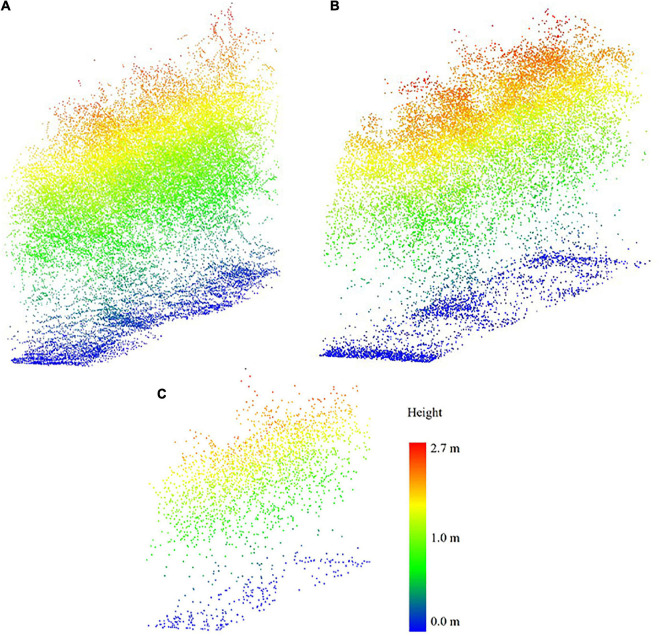
Example sensor point cloud sample data from (7/20/2020) from **(A)** PhenoRover, **(B)** UAV-2, and **(C)** UAV-1.

The average point densities of the LiDAR data acquired by the sensors on the UAVs depend on the type of sensor, the platform flying height, FOV, and mission characteristics such as the sidelap of the flightlines. In this study, point density is investigated based on flying height and sensor type, and it is presumed that the rest of the characteristics affecting point density are consistent across the data acquisitions; these values are significantly lower than the LiDAR point density from the PhenoRover because the sensor on the PhenoRover operates at a much lower height (approximately 5 m from the ground). Table 4 shows the point density of the sensors based on flying height. Figure 5 illustrates the resulting 3D point cloud from the UAV platforms and PhenoRover over a sorghum sample row. As expected, the canopy penetration achieved by the UAV sensors was lower than the PhenoRover due to the higher platform altitude. UAV-2 with a Velodyne VLP-32C had a higher point density, resulting in greater canopy penetration compared to UAV-1 with a Velodyne VLP-Puck LITE, due to the combined impact of being flown at 20 m and the higher pulse rate of the sensor with more laser beams.

**TABLE 4:** Point density of sample data on 7/20/2020.

Platform	Flying height	DAS	Point density (Points/m^2^)
UAV-1	40 m	68	70
UAV-2	20 m	68	500
PhenoRover	N/A	68	1,400

## Methodology

### Feature Extraction From Light Detection and Ranging Data

**FIGURE 6:**
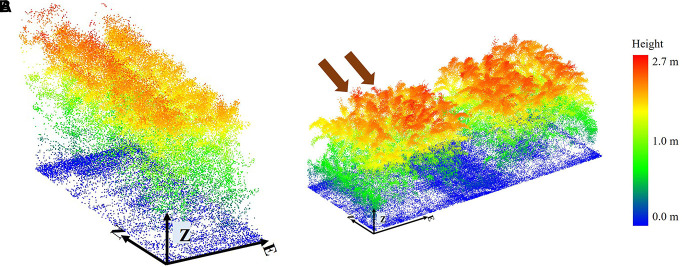
Typical plot; **(A)** HIPS. **(B)** SbDivTc_Cal: rows 2 and 3 selected to extract features. The two arrows indicate rows 2 and 3. The orientation of the plot is shown with arrows (E: Easting, N: Northing, and Z: Elevation).

In the HIPS experiment, LiDAR features were extracted at plot level as there were two rows in a plot (Figure 6A), while in the SbDivTc_Cal experiment, LiDAR features were extracted at the row-level within ten-row plots. Rows four, seven, and eight were adjacent to rows that were destructively sampled. Rows one and ten were “border” rows, so they were not necessarily representative of conditions within the plot, particularly for light accessibility when plots with tall varieties were adjacent to plots with short varieties. Rows 2 and 3 were extracted from the remotely sensed data and analyzed for this study. Features were extracted from rows 2 and 3 as a spatially contiguous two-row block (essentially equivalent to a two-row plot) where the ground reference was collected. Figure 6B shows a typical plot of the dataset, where rows 5 and 6 were destructively sampled via machine harvesting, and manual destructive harvesting was performed in row 9.

**FIGURE 7:**
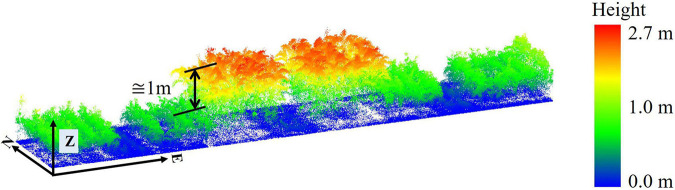
Height of photoperiod sensitive variety SP SS405 FS relative to the surrounding plots 7/28/2020.

Three varieties of sorgum experiment (ATx623xDwfYellMilo, ATx623xSC0044, and SP SS405 FS) are photoperiod sensitive, as noted previously, and have a different plant structure than the rest of the varieties, especially later in the growing season. For example, “SP SS405 FS” was taller than the surrounding plots by approximately 1.3 m on 7/28/2020 (Figure 7). The impact of these varieties on the predictive models was investigated.

As noted in the Introduction, most LiDAR-based features proposed in the literature are based on the height or moments of the histograms of point cloud values in a 3D volume classified as vegetation. The Digital Terrain Model (DTM) required to determine plant heights was derived from a bare earth field using UAV-based LiDAR point cloud data before planting and assumed to be constant throughout the growing season. The height of points was estimated by subtracting the DTM from the “*z*” coordinate of each point in the dataset. Points with a height of less than 10 cm were considered as ground points and not included in the statistical analysis of the vegetation. The following physically-based features were explored for this study.

Laser penetration index is defined as the fraction of laser points that penetrate the canopy. The index can be calculated in many ways. In this study, it is computed as the ratio between the number of ground points (*N*_*Ground*_) and the total number of points in a given area (*N*_Ground_ + *N*_vegetation_), which is assumed here to be a row of a plot (Eq. 2). The number of non-ground points is assumed to be equal to the number of points identified as vegetation (*N*_vegetation_):


(2) $$\text{LPI}:\frac{N_{\text{Ground}}}{N_{\text{Ground}}+N_{\text{vegetation}}}$$

Features commonly used for allometric relationships include various statistically-based height features extracted from the non-ground point cloud, including plant height at various percent quantiles, mean height, standard deviation of the point cloud height, coefficient of variation of height, skewness of height, and Vegetation Complexity Index (VCI) described in Eq. 3 (van Ewijk et al., 2011).


(3) $$\text{VCI}=\frac{\left(-\sum_{i=1}^{\text{HB}}\left[p_{i}\times \ln\left(p_{i}\right)\right]\right)}{\ln\left(\text{HB}\right)}$$

where HB = total number of height bins, *p*_*i*_ = Proportional abundance ($\frac{\#\,\mathrm{of}\,\mathrm{returns}}{\mathrm{Total}\,\#\,\mathrm{of}\,\mathrm{returns}}$) in a height bin (*i*).

**FIGURE 8:**
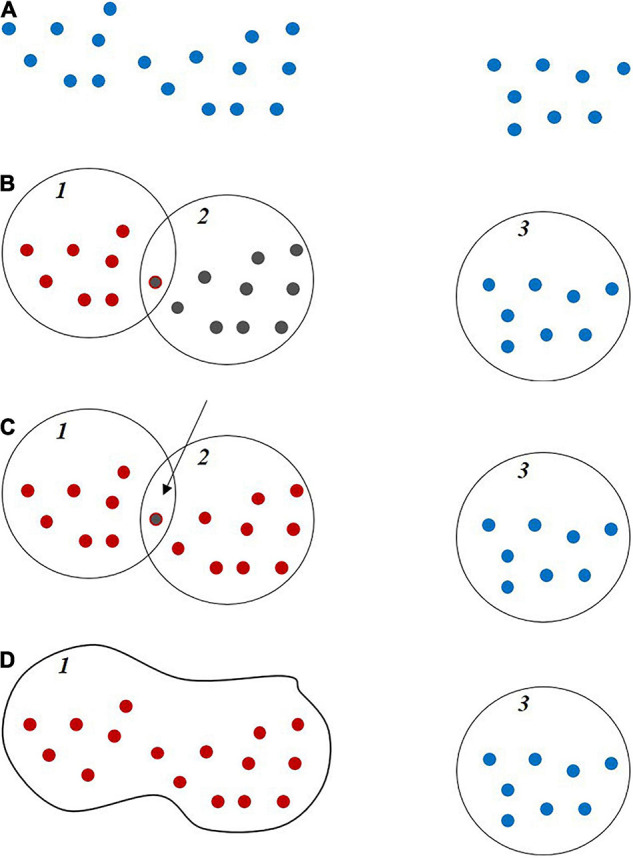
Thematic region growing clustering steps: **(A)** Sample points. **(B)** Initial clustering. **(C)** Finding common points in two close clusters. **(D)** Connecting and joining two clusters.

A new feature, referred to as the Clusters’ Area Plane (CAP), which is based on horizontal characteristics of the point cloud at a given height in a row, was proposed and evaluated in the study. To obtain the CAP feature, a plane is intersected with the point cloud within a row at a given height quartile with ±4 cm thickness of this plate, and the associated points are extracted. The points are clustered using a region-growing approach based on the distance between points and the k-nearest neighbors as follows: the points are represented using a KD tree data structure, and the k-nearest neighbors to each point are determined within a defined radius and assigned to the respective clusters. Then, the clusters with common points are joined, and the cluster number is updated iteratively until no further changes occur in the clusters (Figure 8).

Finally, the area of clusters that is larger than a user-defined threshold is calculated, and the total area is defined as the CAP feature (Eq. 4).


(4) $$\text{CAP}=\sum_{i=1}^{n}A_{i}$$

**FIGURE 9:**
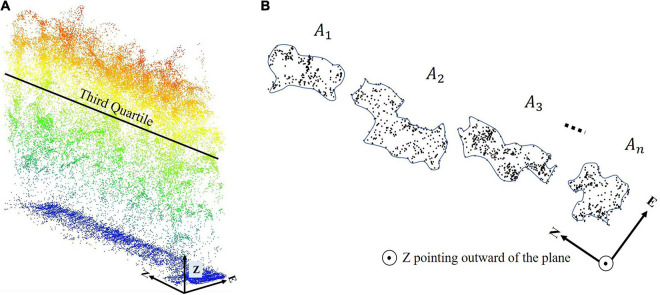
Example of Clusters Area Plane (CAP) feature; **(A)** A typical third quartile of a row and **(B)** cross section at the third quartile.

While the feature does not have a direct physical interpretation, it contains information for predicting LAI based on the horizontal distribution of the plants within the canopy at a given quartile (75% with ±4 cm thickness in this study). The CAP feature was also calculated in other quartiles, e.g., 50% and 25%, but only the 75% quartile provided statistically significant results for the data in these experiments. The 50 and 25% quartiles did not have an adequate number of samples to evaluate the index, both due to penetration of the canopy and its geometric structure. Figures 9A,B show a typical example of the CAP feature.

**FIGURE 10:**
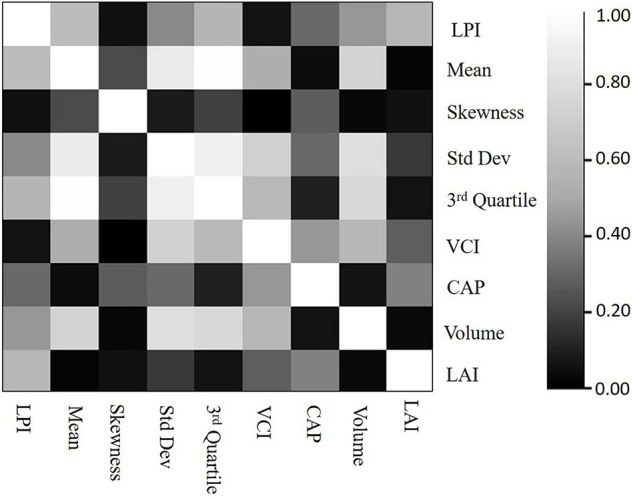
Sensitivity analysis: Feature correlation matrix (zero is the lowest correlation and 1 is the highest correlation).

Correlation between features and LAI indicated that LPI has the highest correlation with LAI, and the CAP feature has the second-highest correlation with LAI. The correlation matrix in Figure 10 also indicates that there is significant correlation between many of the candidate features. For example, the value of the correlation between the standard deviation of height and the mean and third quartile height is greater than 0.9.

### Regression-Based Predictive Models

Predictive models were developed using SMLR (Johnsson, 1992), PLSR (Rosipal and Krämer, 2005), and SVR (Feng and Li, 2014). SVR models were investigated with four kernels (linear, polynomial, RBF, and sigmoid), and their hyperparameters were obtained via grid search. Eight features were considered as input variables, including LPI, Height_mean, standard deviation, and skewness, height (3rd Quartile), VCI, Volume of the vegetation in a row based on the convex hull of the points, and CAP. In this study, the training and test data were selected randomly by 75% training and 25% test. Both replicates of each genotype variety were randomly assigned to either training or test. Ten-fold cross-validation was performed on the training set. The values of *R*^2^ for the respective models are reported in the results section.

## Leaf Area Index Predictive Model Results

**FIGURE 11:**
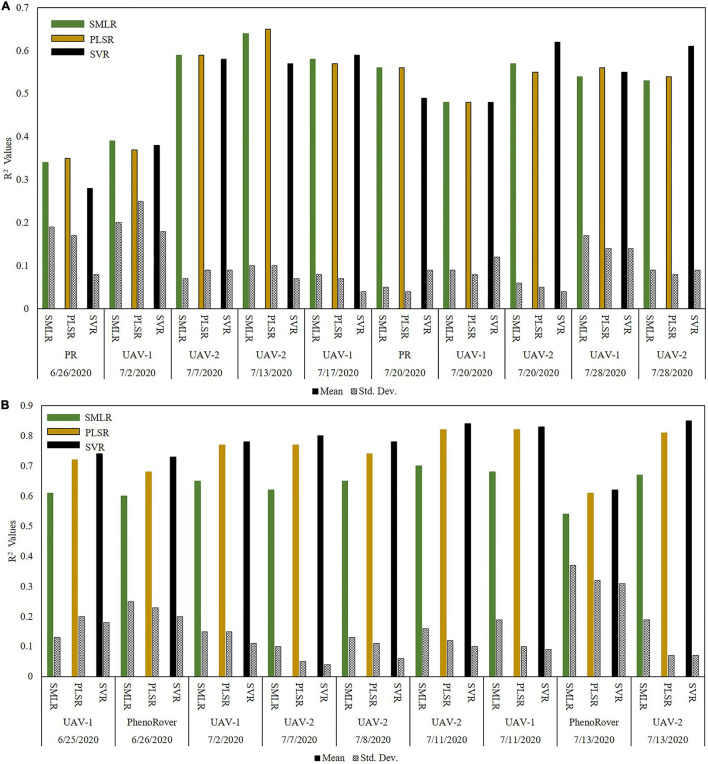
*R*^2^ values for 2020 regression models for LAI estimation **(A)** SbDivTc_Cal and **(B)** HIPS; (PR: PhenoRover).

The results of the LAI predictive models are included based on the date and the platform. SMLR, PLSR, and SVR with RBF kernel models developed for the 2020 sorghum and maize data are illustrated via bar charts. Figure 11A shows the results for sorghum datasets. The models had low *R*^2^ statistics for the first two dates acquired by PhenoRover and UAV-1 (0.28 and 0.38 for the SVR model). The primary reason was the small size of the plants (∼35 and ∼50 cm) for 6/26/2020 and 7/02/2020, respectively. The measurements from the LAI–2200C acquired between the rows were also not representative of the true canopy gap fraction at this height. The values of *R*^2^ for the rest of the dates were consistent throughout the season, even as the plant heights increased rapidly until flowering. Figure 11B shows the results for maize datasets. The values of *R*^2^ for all dates were consistent throughout the season and varied from 0.5 to 0.8. The results of maize show that the range of *R*^2^ in maize is consistant with sorghum, but generally higher. This is attributed to the maize experiment being planted less dense than sorghum (maize: ∼75,000 plants/hectare vs. sorghum: ∼200,000 plants/hectare) and the lower complexity of the plant structure resulting in greater laser penetration into the canopy later in the season. The *p*-value from *t*-test statistics (0.94) showed that differences in the mean of *R*^2^ values from pairwise comparisons of the three regression models were not statistically significant at an α of 0.05. The results also did not indicate significant differences between the mean of *R*^2^ values from pairwise comparisons of combinations of UAV-1 (VLP 16, flown at 40 m), UAV-2 (VLP 32 flown at 20 m), and the PhenoRover.

**FIGURE 12:**
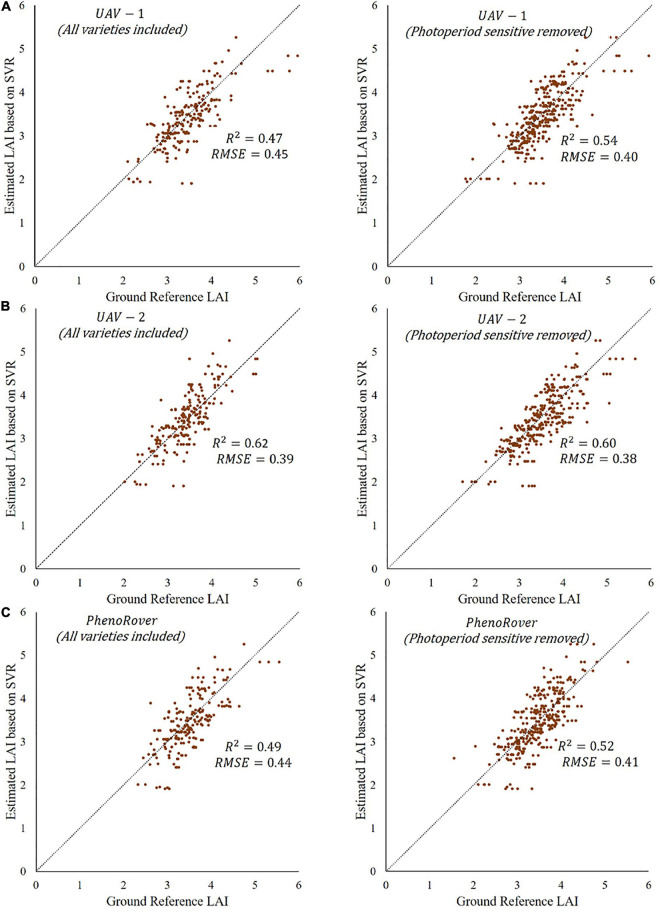
Predictions based on SVR RBF models showing *R*^2^ values and RMSE at midseason (7/20/2020) before and after removing photosensitive varieties for three platforms: **(A)** UAV-1, **(B)** UAV-2, and **(C)** PhenoRover.

The three photoperiod sensitive varieties were removed from the sorghum dataset, and *R*^2^ values of all models were calculated. The *p*-value from *t*-test statistics (0.57) indicated no significant difference between the mean of *R*^2^ obtained using data prior to and after removing photoperiod sensitive varieties. For example, the plots of one-to-one comparisons of reference vs. the predicted values of SVR model from the UAVs and PhenoRover on 7/20/2020 before and after removing the photoperiod sensitive varieties from the datasets are provided in Figure 12. The plots show the model of UAV-1 (Figure 12A) and PhenoRover (Figure 12C) slightly improved in terms of R^2^, but UAV-2 results (Figure 12B) were essentially unchanged.

To evaluate the importance of the features, a leave-one-out procedure was used with the SVR-RBF model, which had the highest *R*^2^ value, and the resulting *R*^2^ (${\text{R}}_{\text{new}}^{2}$) was calculated (Eq. 5),


(5) $$\mathrm{Weight}\,\mathrm{of}\,\mathrm{feature}=1-\frac{R_{\text{new}}^{2}}{R_{\text{original}}^{2}}$$

where $R_{\text{new}}^{2}$ is an *R*^2^ of the model fit without the feature, and $R_{\text{original}}^{2}$ is the *R*^2^ of the model with all features.

**FIGURE 13:**
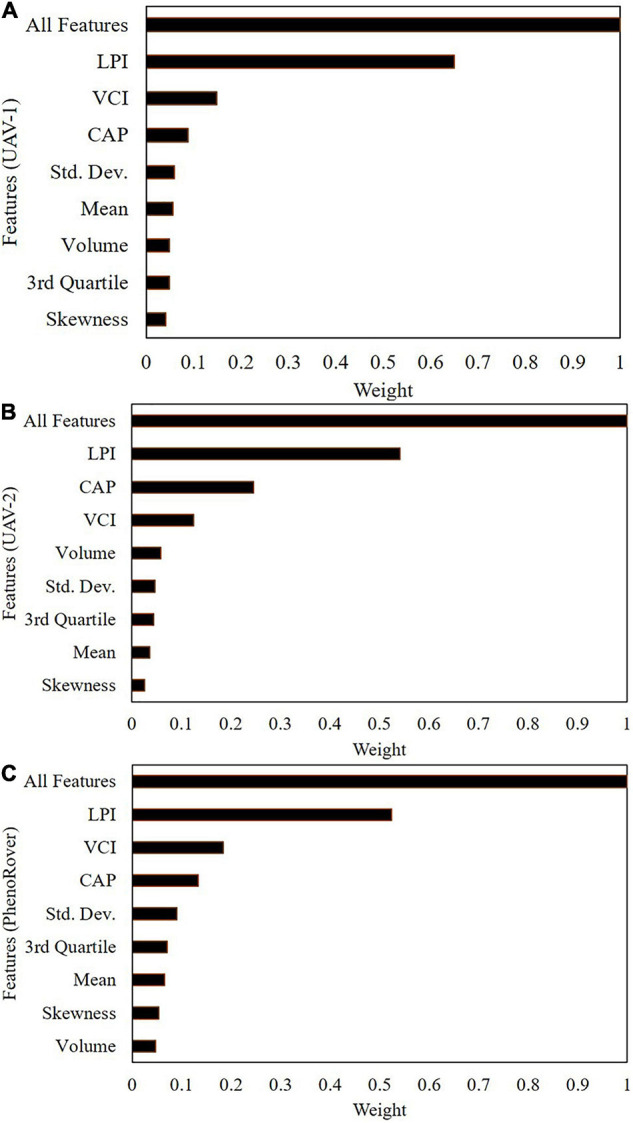
Feature weight evaluation using SVR (RBF) on 7/20/2020: **(A)** UAV-1, **(B)** UAV-2, **(C)** PhenoRover.

Figure 13 shows the feature importance in the models developed for the three platforms on July 20, 2020.

Laser penetration index is the most highly ranked feature, based on the correlation with the plant canopy analyzer data, and the 2nd and 3rd ranked features are CAP and VCI, both of which are also indicative of penetration of the canopy. Additionally, the CAP feature is related to the horizontal distribution of the canopy, as noted previously. The height-related features are correlated and individually have a lower impact on the model, while LPI and CAP represent physically different characteristics. In complex vegetation such as sorghum, which is planted at high density and has tillers, many laser points are concentrated in the upper canopy, and few laser points penetrate deeper in the canopy.

Although the sensor on the PhenoRover was much closer to the canopy, typically between 2 and 5 m depending on the date, and the speed of the PhenoRover was much slower, resulting in increased point density and penetration of the canopy, *R*^2^ values of the models (Figure 11) based on data from PhenoRover, UAV-1 (flying height 40 m) and UAV-2 (flying height 20 m) were similar for comparable dates. In most cases, multiple stepwise linear regression models had the lowest *R*^2^ value, and only LPI and VCI features were significant at α = 0.05. As mentioned earlier, the *R*^2^ value for the SMLR, PLSR, and SVR (with an RBF kernel) models are generally similar, and the sample mean of the *R*^2^ values over the season are not statistically different by pairwise comparison in both sorghum and maize.

## Summary and Conclusion

In this exploratory study, the capability of discrete return LiDAR data was investigated for predicting LAI_eff_. The primary contribution was to develop statistically significant predictive models of LAI over two row crops based on physical features from LiDAR data acquired by multiple platforms during the growing season. In 2020, UAVs and a wheel-based LiDAR dataset were collected and analyzed over two different experiments using a LAI-2200C plant canopy analyzer. The results based on *R*^2^ values indicate that the LiDAR data are capable of estimating LAI after ∼60 DAS. The *R*^2^ results from maize were compatible with the results from sorghum, and somewhat higher due to less dense planting and complexity in canopy geometry. LiDAR data acquired from the UAV-2 with a Velodyne VLP-32C were higher density, and there was greater penetration of the canopy compared to UAV-1 with a Velodyne VLP-Puck LITE. This was due both to the sensor and the lower flight altitude. However, the *R*^2^ values of the resulting models for LAI were not significantly different. This implies either that the relationship to LAI was dominated by the upper canopy structure or that the penetration associated with more beams and lower flying height was not enough greater to impact the models. Additionally, while the lower height of the boom on the PhenoRover platform was expected to provide improved models due to increased density and penetration, the within-canopy scattering and movement of plants by the platform, especially later in the season, were offsetting problems. As the *t*-test showed, differences in the *R*^2^ values of the models obtained for the different platforms and sensors were not statistically significant. In most datasets, the UAV–based models had higher *R*^2^ values than wheel-based data in 2020, especially later in the growing season when the complex scattering between the near range LiDAR and the canopy appeared to impact the models in both sorghum and maize experiments. The inclusion of data from sorghum photoperiod sensitive varieties did not have a significant impact on the results.

The study encountered multiple challenges, including the limitation of acquiring more wheel-based data subject to weather and field conditions throughout the season. The more frequent remote sensing data acquisition and investigation of the plant canopy analyzer data in 2020 were motivated by the need for more frequent data acquisitions during the vegetative stages of the growth cycle when the plants were growing rapidly and during flowering. The LiDAR data were also impacted by multi-path effects because of the complexity of plants associated with plant density and geometry of sorghum. This motivates further research on denoising approaches. In addition, data encoding approaches may prove useful as an alternative to traditional physical structure-based approaches. The study was conducted in a local environmental condition, and the data were acquired under consistent weather conditions. However, the impact of multiple locations, years, different environmental conditions, soil types, and edaphic factors need to be investigated for the robustness of the models in the application of transfer learning. Finally, further studies are also required, including investigation of other sensor modalities and the sensitivity of the various methods in providing ground reference data and their impact on prediction models.

## Data Availability Statement

The raw data supporting the conclusions of this article will be made available by the authors, without undue reservation.

## Author Contributions

BN and MC: conceptualization, formal analysis, and methodology. MC: supervision, writing—review and editing. BN: writing—original draft. MT: writing—review and editing. All authors have read and agreed to the published version of the manuscript.

## Conflict of Interest

The authors declare that the research was conducted in the absence of any commercial or financial relationships that could be construed as a potential conflict of interest.

## Publisher’s Note

All claims expressed in this article are solely those of the authors and do not necessarily represent those of their affiliated organizations, or those of the publisher, the editors and the reviewers. Any product that may be evaluated in this article, or claim that may be made by its manufacturer, is not guaranteed or endorsed by the publisher.
